# Accuracy of transcutaneous bilirubin on covered skin in preterm and term newborns receiving phototherapy using a JM-105 bilirubinometer

**DOI:** 10.1038/s41372-019-0557-9

**Published:** 2019-11-25

**Authors:** U. Costa-Posada, A. Concheiro-Guisán, M. F. Táboas- Ledo, E. González-Colmenero, M. L. González-Durán, M. Suarez-Albo, C. Duran Fernández-Feijoo, M. Pumarada-Prieto, Cristina Martínez-Reglero, J. R. Fernández-Lorenzo

**Affiliations:** 1 Department of Pediatrics, Alvaro Cunqueiro Hospital, Vigo, Spain; 2 Department of Neonatology, Alvaro Cunqueiro Hospital, Vigo, Spain; 3 Department of Methodology and Statitics, Alvaro Cunqueiro Hospital, Vigo, Spain

**Keywords:** Paediatrics, Pain management

## Abstract

**Objective:**

Determine the suitability of transcutaneous bilirubin (TCB) as a tool to assess the effectiveness of phototherapy on patched skin.

**Study design:**

A prospective observational study was conducted. We covered a fragment of skin (sternum) with a photo-opaque patch. Several simultaneous TCB and TSB measurements were performed with the JM-105 bilirubinometer. Bland and Altman test evaluated the agreement between bilirubin levels.

**Result:**

A total of 217 patients were studied, 48.8% were preterm. The mean difference between TSB and TCB before the start of treatment was 1.07 mg/dL. During phototherapy, differences on covered skin were 0.52, 0.27, and 0.39 mg/dL at 24, 48, and 72 h of therapy respectively. The best correlation was observed at 48 h in preterm infants.

**Conclusion:**

The measurement of TCB on patched skin (PTCB) is useful for monitoring the response to phototherapy in term and preterm infants. We use a patch with a removable flap that eases successive measures without disturbing the patients.

## Introduction

Jaundice is a frequent and usually physiological problem in the neonate. However, it can become serious and produce neurological sequelae. Up to 10% of full-term newborns and 25% of preterm develop significant hyperbilirubinemia that requires phototherapy. Patients undergoing phototherapy require frequent monitoring of serum bilirubin levels to monitor response. A noninvasive alternative is the determination of transcutaneous bilirubin (TCB). The new transcutaneous bilirubinometers use multiwavelength spectral reflectance to measure the optical density of cutaneous bilirubin and present greater precision than former devices [1]. Over the years, a close correlation has been demonstrated between TCB and serum bilirubin (TSB), the first is being currently used as a screening method for suspected neonatal jaundice [2]. However, until now, the use of TCB is not recommended once phototherapy has started as the lightening of the exposed skin decreases its reliability [3, 4]. For this reason, some published studies have evaluated whether the measurement of TCB in covered skin (PTCB) could maintain the correlation with TSB. These studies show contradictory results and some of them include a limited number of patients, especially as regards preterm infants [3, 5, 6]. Most of them shared the same model of bilirubinometer, together with a concrete patch, both different to our study's.

The present study was designed with the objective of evaluating the correlation existing between the TCB, measured with a specific bilirubinometer on covered skin with an alternative removable patch, and the TSB during the treatment with phototherapy. We have evaluated if this measurement is affected by either the length of phototherapy or the patient´s gestational age.

## Methods

This study was carried out in the Neonatology Unit of a level III hospital that receives around 3800 births per year. The study population included term and preterm infants (<37 weeks) who met the inclusion criteria: diagnosis of hyperbilirubinemia and indication of treatment with phototherapy during the period of study (June 2016–June 2018).

We established the indication to start phototherapy after Bhutani modified normogram for term and preterm infants. This normogram takes into account TSB values, hours of life, and gestational age to determine the level of hyperbilirubinemia that goes into the range to start phototherapy [2].

**Fig. 1:**
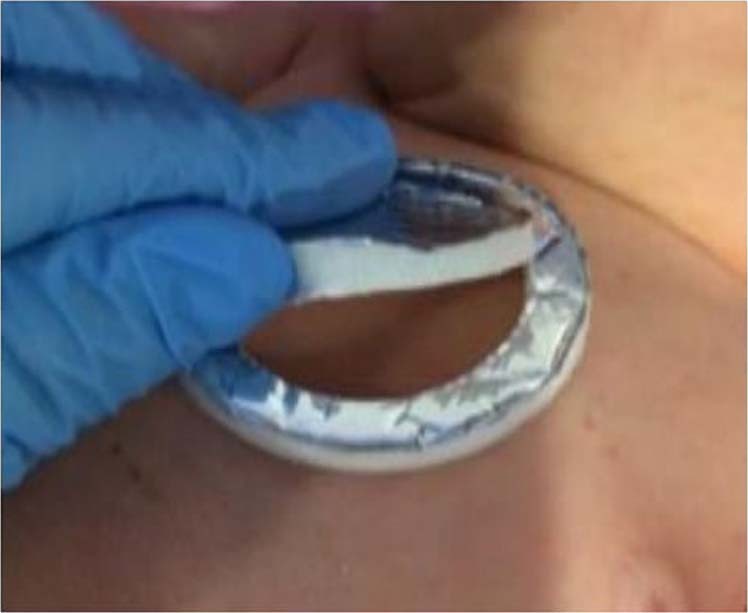
Photo-opaque patch with an easy-removable flap placed at newbornʼs sternum (Informed consent was obtained for this picture)

During the treatment with phototherapy (Lullaby LED Ohmeda medical®/NeoBLUE blanket LED Natus®) the neonates remained naked, with diaper and eye protection. In addition, part of the anterior thorax (sternum) was covered with an opaque photo-reflective aluminum patch (Heat Reflecting Patch, Ohmeda medical®) (Fig. 1) which has a flap that can be easily removed and that is not adhered to the skin of the newborn. In each patient, several sets of simultaneous TCB and TSB measurements were performed. The bilirubinometer used was the model JM-105 (Dräger Medical Systems®).

Prior to the start of phototherapy, a single TCB determination was made at the sternum and, after phototherapy, TCB determinations were made in two locations: on the skin under the patch, and on the exposed naked skin close to it. TSB was determined by diazo-reaction technique. Subsequently, both types of measurements (TCB and TSB) were prospectively collected at 24, 48, and 72 h of treatment.

Informed consent to participate in this study was signed by both parents of every child and approved by our Local Ethics Committee.

Data analysis was performed by the biostatisticians from the Galicia Sur Health Research Institute (IISGS), who employed the SPSS programme (IBM SPSS Statistics 23.0). The method of Bland and Altman was used to evaluate the agreement between bilirubin levels in blood and skin.

## Results

A total of 217 patients were studied, 111 (51.2%) were full-term and 106 (48.8%) were preterm (27–36 + 6 weeks). The demographic and perinatal characteristics of the patients are shown in Table 1. All the patients were Caucasian. Hyperbilirubinemia was considered idiopathic in 82% of infants and related to AB0 isoimmunisation in 18%. The mean age at the beginning of phototherapy was 68.2 h of life and the mean duration of therapy 54.5 h. The mean difference of bilirubin levels with respect to the blood exchange transfusion indication threshold was 6.06 mg/dL (SD 2.82) and no patient in the study did exceed this threshold. Not all patients in the study were subjected to all TCB and TSB determinations because in some cases they finished the therapy earlier or they lacked a simultaneous determination of TSB. For example, only 144 patients out of 217 underwent a simultaneous determination of TSB and TCB before the start of phototherapy. In total, 512 TCB measurements were analyzed, 368 of them during the use of phototherapy and in two locations (covered skin and exposed skin). Table 2 shows the patients distribution during the different times in the study.

**Table 1:** Demographic and perinatal characteristics of our patients

	Preterm neonates *N* = 106 (48.8%)	Term neonates *N* = 111 (51.2%)
Mean weight at birth (g)	2219.81	3182.57
Female (%)	47.2	45.9
Cesarean section (%)	35.8	9
Intensive care provided (%)	38.7	9.9
Weight loss since birth to the time phototerapy was started (%)	4.46	3.3

**Table 2:** Distribution of all patients (*N*) along the time of the study

Number of patients (*N*)	Pretreatment	After 24 h of photherapy	After 48 h of photherapy	After 72 h of photherapy
Term	90 pt	54 pt	31 pt	16 pt
Preterm	54 pt	54 pt	19 pt	10 pt
Total	144 pt	108 pt	50 pt	26 pt

**Fig. 2:**
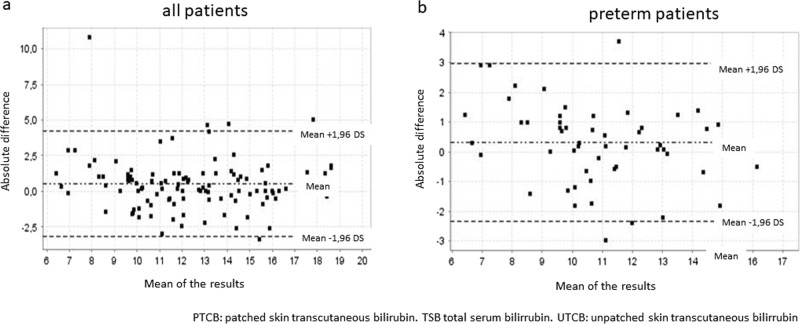
Bland–Altman plots. Agreement between PTCB and TSB after 24 h of phototherapy, (**a**) total (**b**) preterm. The *x*-axis shows the mean of the results ([TSB + PTCB]/2; [TSB + UTCB]/2) of the two methods. The *y*-axis shows the absolute difference between the two methods (TSB–PTCB; TSB–UTCB)

**Fig. 3:**
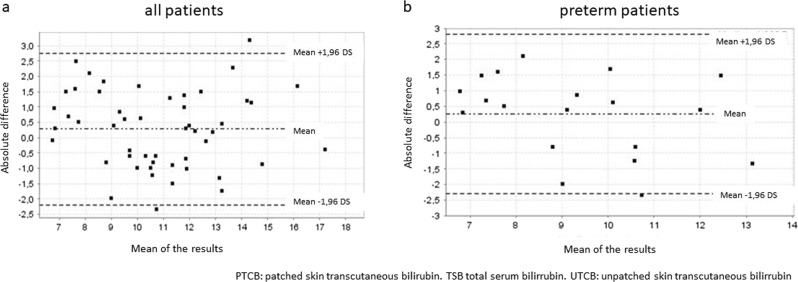
Bland–Altman plots. Agreement between PTCB and TSB after 48 h of phototherapy, (**a**) total (**b**) preterm. The *x*-axis shows the mean of the results ([TSB + PTCB]/2; [TSB + UTCB]/2) of the two methods. The *y*-axis shows the absolute difference between the two methods (TSB–PTCB; TSB–UTCB)

In Tables 3–5 we present our main findings according to the mean of differences between TCB and TSB obtained at each time interval of the study for term and preterm patients. Figures 2 and 3 show Bland–Altman plots after 24 and 48 h of phototherapy, respectively.

**Table 3:** Mean of the differences between UTCB or PTCB (0, 24, 48, and 72 h) and TSB, SD, and IQ range 95% for term infants

Phototherapy length	UTCB-TSB	PTCB-TSB
0 h	1.33 mg/dl (SD 1.84) IQ range 95%: 0.95–1.72	------------------------------------
24 h	8.69 mg/dl (SD 3.03) IQ range 95%: 7.68–9.70	0.74 mg/dl (SD 2.29) IQ range 95%: 0.11–1.37
48 h	6.94 mg/dl (SD 3.19) IQ range 95%: 5.29–8.58	0.29 mg/dl (SD 1.26) IQ range 95%: −0.16–0.76
72 h	8.31 mg/dl (SD 1.88) IQ range 95%: 6.74–9.88	0.75 mg/dl (SD 1.76) IQ range 95%: −0.19–1.69

Mean of the differences between UTCB or PTCB (0, 24, 48, and 72  h) and TSB, SD, and IQ range 95% for term infants

*UTCB* transcutaneous bilirubin on naked skin, *PTCB* transcutaneous bilirubin on patched skin, *TSB* serum bilirubin, *SD* standard deviation *IQ* interquartile

**Table 4:** Mean of the differences between UTCB or PTCB (0, 24, 48, and 72 h) and TSB, SD, and IQ range 95% for preterm infants

Phototherapy length	UTCB-TSB	PTCB-TSB
0 h	0.64 mg/dl (SD 1.84) IQ range 95%: 0.13–1.14	----------------------------------
24 h	6.33 mg/dl (SD 2.72) IQ range 95%: 5.02–7.64	0.29 mg/dl (SD 1.35) IQ range 95%: 0.29–0.66
48 h	5.61 mg/dl (SD 2.53) IQ range 95%: −0.66–11.89	0.24 mg/dl (SD 1.46) IQ range 95%: −0.38–0.87
72 h	6.32 mg/dl (SD 1.94) IQ range 95%:−11.14–23.79	−0.18 mg/dl (SD 1.76). IQ range 95%: −0.82–0.46

*UTCB* transcutaneous bilirubin on naked skin, *PTCB* transcutaneous bilirubin on patched skin, *TSB* serum bilirubin, *SD* standard deviation, *IQ* interquartile

**Table 5:** Mean of the differences between UTCB or PTCB (0, 24, 48, and 72 h) and TSB, SD, and IQ range 95% for all infants

Phototherapy length	UTCB-TSB	PTCB-TSB
0 h	1.07 mg/dl (SD 1.86) IQ range 95%: 0.77–1.38	----------------------------------
24 h	7.89 mg/dl (SD 3.11) IQ range 95%: 7.05–8.73	0.52 mg/dl (SD 1.88) IQ range 95%: 0.16–0.88
48 h	6.74 mg/dl (SD 3.08) IQ range 95%: 5.30–8.18	0.27 mg/dl (SD 1.26) IQ range 95%: −0.08–0.63
72 h	7.91 mg/dl (SD 1.97) IQ range 95%: 6.50–9.32	0.39 mg/dl (SD 1.54) IQ range 95%: −0.23–1.01

*UTCB* transcutaneous bilirubin on naked skin, *PTCB* transcutaneous bilirubin on patched skin, *TSB* serum bilirubin *SD* standard deviation, *IQ* interquartile

**Fig. 4:**
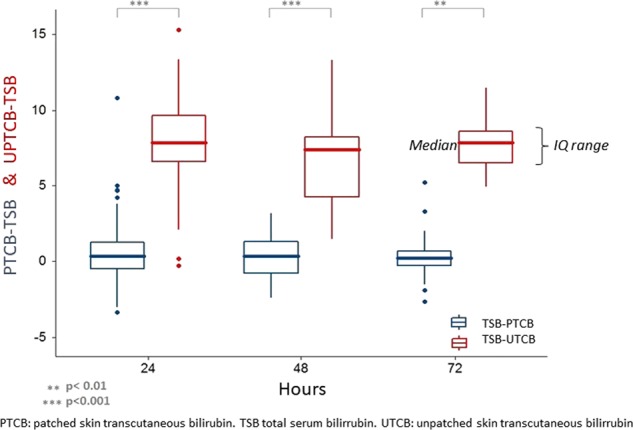
Graph depicting the difference between TCB on patched skin (PTCB) or unpatched skin (UTCB) and TSB at various time moments (hours of phototherapy). Mean of differences and interquartile range

In summary, we have found a good correlation between TSB and TCB before the start of treatment. After the initiation of phototherapy, the good correlation between TSB and TCB continued for measurements on skin covered with a patch at 24, 48, and 72 h of therapy, both for preterm and term children. However, a significant loss of reliability of TCB on uncovered skin was observed. The difference between TCB on exposed and unexposed skin with respect to TSB reached statistical significance at 24 and 48 h (*p* < 0.001) and at 72 h (*p* < 0.01) (Fig. 4). Maximum agreement was reached at 48 h (Figs. 2–4, Tables 3–5). The correlation was better in preterm infants. The agreement remained stable at hyperbilirubinemia levels, even those above 15 mg/dL. None of the patient’s perinatal characteristics (Table 1) influence the degree of correlation between TCB and TSB.

## Discussion

To date, after the study by Zecca et al. [7], this is the largest series of patients published using TCB with a photo-skin coverage system. This is also the series with the largest number of TCB determinations recorded on covered skin and the second in terms of the number of preterm infants included. Considering that the majority of studies using a skin cover have been performed with the Bilicheck® device [5–13], our series is the most representative in number of cases of any gestational age and preterm age using the JM-105 device [14].

The use of TCB as a screening method for neonatal hyperbilirubinemia is included in clinical practice guidelines. The American Academy of Pediatrics recommends it as a screening system in neonates >35 weeks of gestational age [4, 15]. In 2000, Bhutani et al. conducted a study enrolling almost 500 infants which showed the efficacy of TCB in predicting the risk of severe hyperbilirubinemia and the indication of phototherapy [2]. In our work we have obtained adequate results about the usefulness of TCB as a screening tool, similar to those already described [5, 7–9, 16, 17]. The good correlation is maintained in the preterm group, as Nagar’s review (22 studies) had stated, while suggesting a better accuracy with the bilirubinometer JM-103 compared with other devices [18].

Tan and Dong observed that the beginning of phototherapy reduced the correlation of TCB with TSB in skin exposed to light [19]. This fact has been confirmed by other authors [7, 16, 20, 21]. A systematic review and meta-analysis conducted in 2016 exposes the dubious utility of TCB in skin exposed to phototherapy [4]. Our study has also shown a significant decrease in the correlation between TCB and TSB in light exposed skin, with mean differences exceeding 6–8 points. This happens even in preterm patients, contrary to that affirmed by other authors [5, 17].

On the usefulness of covering the skin from exposure to phototherapy, in 1983, Hegyi et al. showed that only the TCB measured in uncovered skin changes during phototherapy, while measurements on covered skin continue to predict the TSB with sufficient reliability [20]. Other subsequent studies carried out with the Bilicheck® bilirubinometer corroborated this statement [5–8, 22]. Zecca et al. [7] used a photo-opaque patch of 2.5 cm in diameter designed for treatment during phototherapy (BiliEclipse, Respironics®) located on newborn´s forehead. They observed that TCB had a slight tendency to overestimate TSB when bilirubin levels were high and that TCB started to underestimate TSB after 96 h of treatment, but in general the correlation shown was good and similar to our findings. Other authors obtained similar results using the same device [5, 6, 8]. However, in a study published in 2017 by Murli performed in 100 newborns, they found a bad correlation between the TSB and TCB before, during and after the treatment [10]. They also used the Bilicheck® but performed TCB measurements in a different area of the skin (sternum) covered by the BiliEclipse® patch.

Regarding our bilirrubinometer, JM-105, or its previous model JM-103, there are scarce studies that have evaluated the usefulness of TCB in covered skin in term children. The prospective study conducted by Casnocha with the JM-103 made determinations only after the end of phototherapy. The reliability of the TCB was low, despite being measured on the unexposed skin (abdomen covered with the diaper), although higher than in exposed areas [3]. Perhaps the good results obtained in our study are related to the place used for the measurements on covered skin (sternum) recommended by the manufacturer.

With respect to the use of TCB on covered skin during phototherapy in preterm infants the studies show contradictory results. In the study by Zecca [7] they found a better correlation in premature than in term infants, like we have done. Similar results are those found by Pendse et al. [13] who employed a methodology similar to ours (aluminum photoprotection system and JM-105). These authors highlighted better results in children under 32 weeks. On the contrary, in the work carried out by Radfar with Bilicheck® they found a worse correlation in the preterm group [5].

In terms of extremely low birth weight premature babies (EBLW), De Luca and Dell’Orto [11] obtained readings in the forehead on skin covered by the cap of the CPAP, and found a good correlation after 4–6 h from the start of phototherapy, with a tendency towards overestimation at high levels of bilirubin. In another study carried out by Bhargava [12], they employed the BiliEclipse® patch coverage, but measurements were made on the back. They observed a tendency to overestimation in the TCB determinations performed on covered skin and to underestimation in the determinations made on skin exposed to light.

Regarding the most premature patients there is controversy about the suitability of placing a patch (given the skin fragility) or not. There are authors such as Cucuy or Roshsiwatmo, who found a good correlation between TSB and TCB on exposed skin measured with the JM-103 bilirubinometer in the sternum, after the initiation of phototherapy in preterm infants [17, 23]. Perhaps the immaturity of the cutaneous barrier of the very premature and its dermal kinetics influences these results, favouring that the reading in uncovered skin is more similar to the TSB. Our EBLW sample is too small to draw conclusions. All results are limited to Caucasian race neonates.

In our series the good correlation between TSB and PTCB improves as the treatment time progresses, reaching the maximum correlation (0.27 mg/dL) at 48 h, showing the usefulness of this technology for the follow-up during phototherapy. The best agreement as the hours of treatment and postnatal age increase could be related to the balance established between the TSB and the TCB in the skin with time. This effect has been observed in term and preterm children.

Finally, in relation to clinical application of our findings, we note that the differences in serum and transcutaneous values in covered skin barely exceed two points of difference in the most unfavorable and infrequent scenario. Therefore it can be recommended as a follow-up method during treatment, with the advantage of fewer blood tests. However, it would be advisable to confirm the value of TCB with a blood test in those cases in which a difference of two points in the bilirubin value implies a change in the therapeutic attitude.
